# Clinical testing of the cardiovascular effects of e-cigarette substitution for smoking: a living systematic review

**DOI:** 10.1007/s11739-022-03161-z

**Published:** 2023-01-07

**Authors:** Giusy La Rosa, Robin Vernooij, Maria Qureshi, Riccardo Polosa, Renée O’Leary

**Affiliations:** 1 Department of Clinical and Experimental Medicine, University of Catania, Catania, Italy; 2 Present Address: Department of Nephrology and Hypertension, University Medical Center Utrecht, Utrecht, The Netherlands; 3 Present Address: Julius Center for Health Sciences and Primary Care, University Medical Center Utrecht, Utrecht University, Utrecht, The Netherlands; 4 Present Address: Center for the Acceleration of Harm Reduction, University of Catania, Catania, Italy; 5 Center for Excellence for the Acceleration of Harm Reduction, University of Catania, Via Santa Sofia, 89 Torre Biologica 11 Piano, 95123 Catania, Italy

**Keywords:** E-cigarette, Harm reduction, Heart rate, Blood pressure, Living systematic review, Risk of bias, ENDS

## Abstract

Some persons who smoke have substituted e-cigarettes for tobacco cigarettes, either completely or partially. What effect does this have on cardiovascular functioning? We conducted a living systematic review on human clinical studies measuring the cardiovascular effects of e-cigarette substitution for smoking. The Scopus, PubMed, and CENTRAL Cochrane Library databases were searched on January 31 and April 29, 2021. Three secondary searches and a grey literature search were conducted. Included study designs were randomized controlled trials, quasi-experimental clinical trials, and cohort studies. Risk of bias and study quality were evaluated with the JBI Critical Appraisal tools and the Oxford *Catalogue of Bias*. The systematic review covered 25 studies comprising 1810 participants who smoked. Twenty studies were rated at high risk of bias, and five as some concerns. A tabular synthesis by direction of effect was conducted due to heterogeneity in the data. Nearly two-thirds of the test analyses indicated that e-cigarette use had no significance difference compared with tobacco cigarettes on heart rate, blood pressure, and in other cardiovascular tests. In two studies, participants with hypertension experienced a clinically relevant reduction in systolic blood pressure after 1 year of e-cigarette use. E-cigarette substitution incurs no additional cardiovascular risks, and some possible benefits may be obtained, but the evidence is of low to very low certainty. An update search on May 30, 2022 retrieved five studies that did not alter our conclusion.

*Registration* PROSPERO #CRD42021239094.

**Supplementary Information:**

The online version contains supplementary material available at 10.1007/s11739-022-03161-z.

## Introduction

More than 1 billion people worldwide are tobacco users, causing more than 7 million deaths annually [1]. For cardiovascular diseases (CVD), smoking is recognized as one of the principal acquired risk factors for atherosclerotic diseases and contributes to the disease burden of aortic aneurysm (34.6%), peripheral artery disease (26.8%), ischemic heart disease (18.41%), and stroke (14.2%) [2]. Two elements of cigarette smoking contribute to causing CVD: the smoke from combustion and nicotine.

Some researchers, particularly those supporting tobacco harm reduction, hold the position that “most of the harm caused by tobacco use is derived from exposure to combustion products of tobacco” [3]. Others disagree, “the relative contributions of nicotine versus non-nicotine components of TC [tobacco cigarette] smoke are unknown” [4]. The effects of inhaled nicotine are difficult to isolate from the smoke constituents (oral nicotine delivery has been studied) and “understanding the role of nicotine in cardiopulmonary disease is extraordinarily difficult” [5]. Research has shown that nicotine activates the sympathetic nervous system, constricting coronary arteries, reducing coronary blood flow reserve, and causing transient increases in heart rate, blood pressure, and myocardial contractability [5–7].

Although smoking risks are well known, cigarette smoking remains widespread. Cigarette smoking is hard to quit. The success rate for cessation is abysmally low, approximately 7% at 6 months [8, 9]. Moreover, adequate tobacco cessation services are not available in many countries [10]. Also, often overlooked, are the pleasurable effects of nicotine [11, 12] that contribute to habituation.

Some people who smoke have tried or are using electronic nicotine delivery systems (ENDS), popularly known as e-cigarettes. ENDS consumption has increased noticeably in the few last years [13]. ENDS may function as a substitute for those who are unwilling or unable to quit [14, 15]. The expected benefits come from the significantly lower exposure to toxicants in ENDS vapor compared to cigarette smoke, including the absence of carbon monoxide [3, 4, 7, 16, 17]. Knowing the health effects of ENDS compared to continued smoking is of primary importance for clinicians and users [18].

Our aim was to conduct a living systematic review with evidence from clinical research on human participants to address the question: “What are the cardiovascular health effects resulting from the substitution of ENDS for conventional cigarettes?”.

## Methods

Our research question was framed with the PICO (population, intervention, comparator, outcome) model.

- Population: adults who smoke cigarettes.
- Intervention: substitution of ENDS for tobacco cigarettes (TC).
- Comparator: participants who continued to smoke or within-subject changes for participants who substituted ENDS for smoking.
- Outcomes: measures of cardiovascular function including blood pressure, heart rate, and other cardiovascular tests.

Our review conforms to the Preferred Reporting Items for Systematic Reviews and Meta-Analysis (PRISMA) 2020 guidelines [19], see Online Resource 1 PRISMA 2020 Checklist. The protocol was registered with PROSPERO #CRD42021239094 and published in a peer-reviewed journal [20].

The baseline literature search was conducted on January 31, 2021 and updated through April 29, 2021. The databases searched were Scopus, PUBMED, and CENTRAL Cochrane Library with the start date of 2010. The search syntaxes are displayed in Online Resource 2 Search Syntax. Reference lists of systematic and narrative reviews on the cardiovascular effects of ENDS use published from 2018 through 2020 were examined for additional studies. Citation chasing of the included studies was conducted in Google Scholar. A grey literature search checked 41 cardiovascular medical organizations (Online Resource 3 Grey Literature Searches).

An update (“living”) search was conducted on May 22, 2022 with the start date of March 2021 to allow for indexing lag. The update search was conducted in the PubMed and Scopus databases. Newly published systematic reviews were checked for additional studies. The grey literature search of medical organizations was not performed. The study selection processes of title/abstract review and full paper were conducted with the same procedures as the baseline search. The reference lists of included studies were checked for additional studies.

Study designs included were human subject randomized and non-randomized controlled trials, clinical trials, prospective and retrospective cohort studies, and case-controlled studies. The first process was the exclusion of articles based on titles and abstracts to remove in vitro and animal studies, commentary articles, and false retrievals. Details of the exclusion criteria are available in the published protocol [20].

The second process for study inclusion was a full paper review. Three inclusion criteria were applied. One, studies were limited to the research designs listed above. Two, a study was required to have either a comparator group who smoked combustible tobacco (cigarettes) or a within-subject testing of participants who had substituted ENDS for smoking. Third, the study had to report outcome data or analysis from a cardiovascular test. All three criteria had to be satisfied for a study to be included. The inclusion and exclusion of studies was conducted independently by two reviewers, and discrepancies were resolved by discussion. There were no unresolved disagreements for the title/abstract sorting, and for full paper review, one study was decided by the Project Leader (RO), 95% inter-rater agreement. For the update search, there were no unresolved disagreements on the title/abstract sorting or the full paper review.

The data extraction process was conducted independently by two reviewers with a pre-specified data extraction form drawn from the JBI Manual [21] and the Cochrane Collaboration Handbook [22]. (Online Resource 4, Data Extraction Form). When published data were insufficient or missing, the corresponding author was contacted via email.

The quality of studies was assessed by two independent reviewers applying the JBI quality assessment tools [21] and a report of biases drawn from the Oxford Centre for Evidence Based Medicine *Catalogue of Bias* [23] (Online Resource 5, Bias Report Form). Discrepancies were resolved by discussion; no third person arbitration was required.

The overall rating of the risk of bias for each study was assessed with a rubric that consisted of the JBI score and the biases reports (Online Resource 6, Bias Rating Rubric). Studies were rated as one of three classifications from the Cochrane handbook [22]: low risk of bias, some concerns, and high risk of bias. The rater (RO) was blinded to study outcomes and funders, and the study bias rating was endorsed by the team members.

In addition, the two reviewers independently completed two quality checks for each study. One report noted deviations from protocol (Online Resource 7, Protocol Deviations Report). Another checklist (Online Resource 8, Data discrepancies) identified discrepancies in the reporting of data and statistical significance with the methodology developed by Puljak et al. [24].

All the reports for data extraction and quality assessment are available in the Zenodo data repository: https://zenodo.org/record/4835883#.YPnMqECxUuU.

Per protocol, a tabular synthesis was conducted on test measurements and a narrative summary was compiled of study biases. As anticipated, no meta-analysis was conducted due to the heterogeneity between the studies. These differences across studies included the ENDS nicotine strength and differing ENDS models, wide disparities in study populations in their smoking history and patterns of use, and differing clinical test parameters (acute and widely differing follow-up periods). Furthermore, the high risk of bias in 80% of the studies precluded conducting a meta-analysis [22, 25].

Six analyses were conducted. One sensitivity analyses excluded all studies at high risk of bias. Another analysis was for effect modifications on findings. Three sub-group analyses were conducted for (1) concurrent users of cigarettes (dual users), (2) participants with prior disease conditions, and (3) ENDS use of a duration of 1 year or longer. Finally, the certainty of evidence was evaluated with Grading of Recommendations Assessment, Development and Evaluation (GRADE) [26].

There were a few deviations from our protocol. Due to the length of the review, we excluded the narrative summary of the individual studies. A sensitivity analysis for conflict of interest for industry studies was not conducted, because all industry studies were at high risk of bias. An analysis of effect modifications was added. Because no meta-analysis was conducted, a formal assessment of publication bias could not be performed. The synthesis of the test tabulations as Vote Counting Direction of Effect was added after data collection and before the start of the analysis. This method categorizes the test outcome as showing benefit (improvement in function), harm (decrease in function), or no difference (no significant effect) as a standardized metric that is counted and compared [22].

## Results

### Study inclusion

**Fig. 1:**
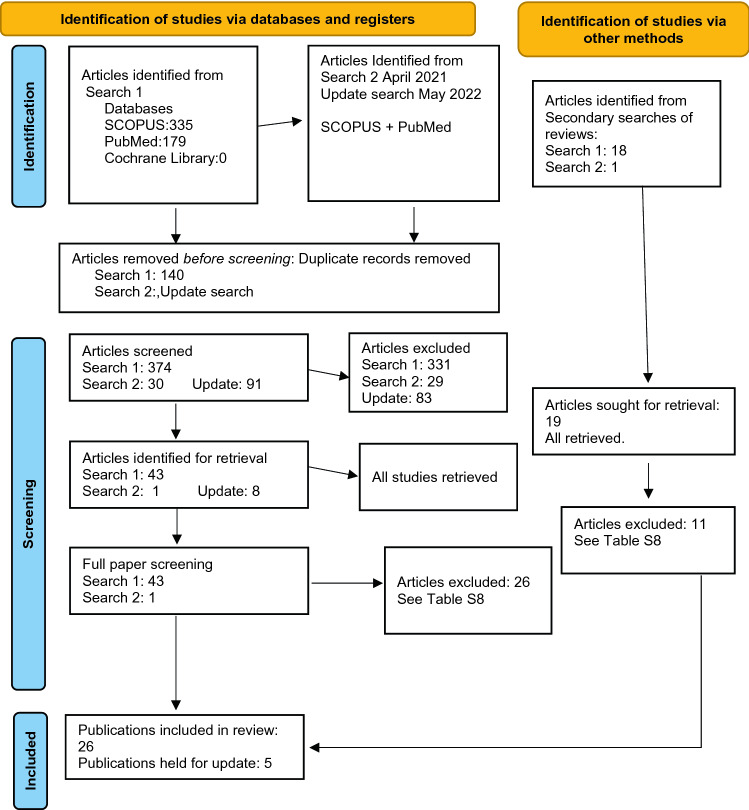
PRISMA flow diagram

The search results are reported in Fig. 1 PRISMA Search Diagram. Publications excluded at full paper review with the reason for exclusion are listed in Online Resource 9, Excluded Studies.

Our baseline systematic review retrieved 25 studies with 26 publications [27–52]; see Table 1, studies included. No grey literature was found.

**Table 1:** Studies included

Study	Design	*N*	Tests	Duration	Bias rating
Biondi-Zoccai [27]	RCT cross-over	20	BP, MPB, FMD	Acute	High
Carnevale [28] Mastrangeli [29]	Quasi-experimental	20	FMD	Acute	Some concerns
Chaumont [30]	RCT	20	HR	Acute	High
Cioe [31]	Quasi-experimental	19	HR, BP	8 weeks	High
Cobb [32]	Quasi-experimental cross-over	20	HR, BP	Acute	High
Cravo [33]^a^	RCT	408	HR, BP, ECG	12 weeks	High
D’Ruiz [34]^a^	RCT	105	HR, BP	5 days	High
Farsalinos [35]	RCT	183	HR, BP	52 weeks	Some concerns
Frazen [36]	RCT cross-over	15	BP, HR	Acute	High
George [37]	RCT	114	HR, BP, FMD	4 weeks	High
Hickling [38]	Quasi-experimental	40	HR, BP	Data at 6 weeks	High
Hiler [39]	RCT	64	HR	Acute	High
Ikonomidis [40]	RCT	40	BP	4 months	Some concerns
Kerr [41]	RCT cross-over	20	HR, BP	Acute	High
Kuntic [42]	Quasi-experimental	20	HR, FMD	Acute	High
Nides [43]^a^	Quasi-experimental	25	HR	Acute	High
Polosa [44]	Cohort	89	HR, BP	12 months	Some concerns
Sumartiningsih [45]	RCT cross-over	24	HR, BP	Acute	High
Szoltysek-Boldys [46]	Quasi-experimental cross-over	15	HR, BP	Acute	High
Van Staden [47]^a^	Quasi-experimental	13	HR, BP	2 weeks	High
Vansickel [48]	Quasi-experimental cross-over	32	HR	Acute	High
Veldheer [49]	RCT	191	HR, BP	3 months	Some concerns
Walele [50]^a^	RCT	12	HR, BP, ECG	Acute	High
Walele [51]^a^	Quasi-experimental	102	HR, BP, ECG	24 months	High
Yan [52]^a^	RCT cross-over	23	HR, BP	Acute	High

*BP* blood pressure, *ECG* electrocardiogram, *FMD* flow-mediate dilation, *HR* heart rate, *RCT* randomized controlled trial

^a^Industry-funded

The data for the tabular test synthesis and study details are reported in Online Resource 10, Study Evidence Table. The studies were conducted in USA (8), UK (6), Italy (4), Germany (2), and one each from Belgium, Greece, Indonesia, Poland, and South Africa. The participants ranged in age from 18 to 65 comprising 1810 participants who smoked. Four studies were conducted with participants with comorbidities of serious mental illness (1), HIV-positive (1), or hypertension (2). Fourteen studies conducted acute testing; 11 studies presented follow-up data ranging from 5 days to 24 months. Study designs were 14 randomized controlled trials (RCT), 10 quasi-experimental (clinical trials), and 1 cohort study. Twenty studies were rated at high risk of bias, five were rated as some concerns, and no studies were rated as at low risk of bias. The JBI assessment scores, methodological issues, and reporting biases are presented in Online Resource 11, Study Biases.

Five recently published studies were retrieved with the update review; see Fig. 1. One follow-up study reported no significant changes in blood pressure with ENDS use [53], two acute studies found no differences in blood pressure or heart rate between ENDS and TC users [54, 55], and a third acute study reported a higher resting heart rate with tobacco smoking compared to ENDS [56]. The fifth study conducted positron emission tomography/magnetic resonance imaging in matched groups of tobacco users and ENDS users aged 18–30 years; they found no evidence of vascular inflammation [57]. As per protocol, because the findings of these studies did not alter our conclusions, the studies will be incorporated into the updated version of the living systematic review planned for the end of 2024.

### Tabular synthesis of cardiovascular tests

**Fig. 2:**
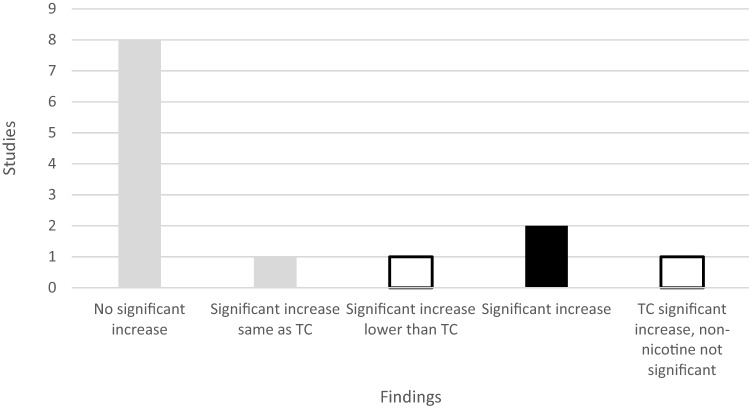
Testing outcomes, acute heart rate

Twenty-two studies tested heart rate (HR), 12 with acute testing and 10 with follow-up (one study data not reported [38]). Acute testing both within-subject and comparing ENDS vs. TC yielded mixed findings; see Fig. 2. Eight acute studies found no significant increase in HR with ENDS use within subject [43, 46, 48, 50] or compared to TC [32, 36, 48, 52]. Three acute tests found a significant increase in HR for nicotine ENDS [39, 41, 43], but in one test, the increase was not significantly different than TC [39], and in another, it was a significantly lower increase than TC [41]. One additional acute study finding an increase in HR was seriously compromised due to excessive ENDS exposure [42]. One study reported that acute non-nicotine ENDS use significantly raised HR compared to sham vaping; it conducted excessive ENDS exposure [30]. Another study on non-nicotine ENDS found that TC significantly raised HR in comparison [45].

**Fig. 3:**
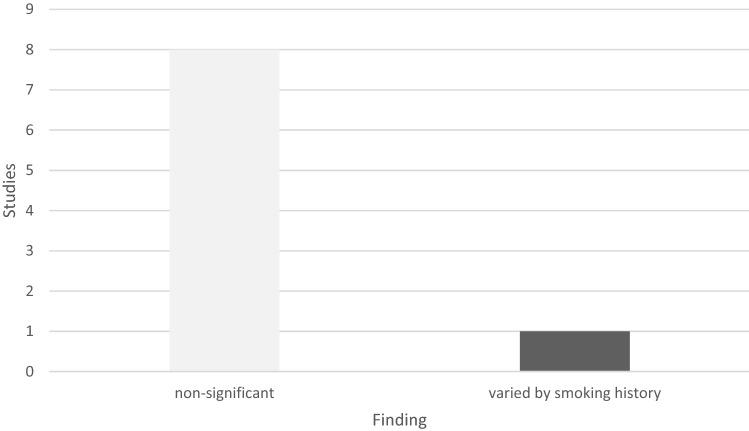
Testing outcomes, follow-up heart rate

Eight of the nine follow-up studies demonstrated no significant changes in HR [31, 33–35, 44, 47, 49, 51], while one study reported differing outcomes based on the participants’ pack year smoking history [37]; see Fig. 3.

**Fig. 4:**
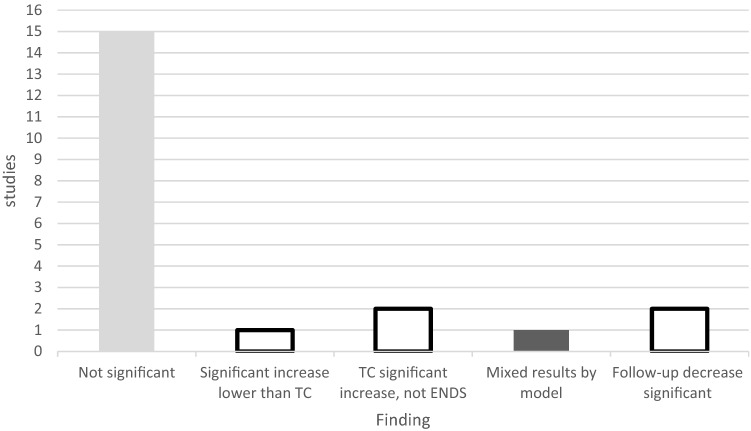
Testing outcomes, blood pressure

Nineteen studies tested blood pressure (BP), 8 acute studies and 11 follow-up studies. Fifteen studies indicated no significant changes [31–34, 36–38, 40, 41, 45–47, 49–51]. One acute test found a significant increase in BP, but the increase was not significantly different from TC, and ENDS increases were lower than TC [27]. Two studies showed that TC significantly increased BP, while ENDS did not [41, 45]. One study with five ENDS models found that some significantly increased BP, while others did not, but the finding is compromised due to excessive ENDS exposure [52]. Two follow-up studies found a significant decrease in BP with ENDS use [35, 44]; see Fig. 4.

Four studies assessed the impact of ENDS on arterial stiffness with flow-mediated dilation (FMD) tests. Two acute studies reported significant declines for within-subject measurements, but the difference between ENDS and TC was not significant and the reduction with ENDS was less than TC [27, 28]. One acute study [42] found significant decreases within subject, but the findings were compromised due to excessive ENDS exposure. A fourth study with a 4-week follow-up calculated that females but not males using ENDS had a smaller decrease than participants continuing smoking [37].

Electrocardiogram (ECG) 12-lead testing was conducted in three studies. No significant findings were reported in acute testing [50] or in the follow-up studies [33, 51].

### Analyses

A sensitivity analysis was made with the five studies at some risk of bias. Their tests results were in much the same ratios as the studies at high risk of bias.

An assessment of effect modification was performed for age, gender, and smoking history. Only one study had a significant result for females but not males, and observed differing results based on participants’ smoking history. No effect modifications were observed in any other studies.

Three sub-group analyses were calculated. For dual users, only one study compared dual users with exclusive ENDS users; finding that exclusive users had significantly better improvements in HR and BP than dual users [44]. For participants with comorbidities, no significant cardiovascular changes were reported for those with HIV [31] or participants with serious mental illnesses [38]. Only two studies were conducted with participants with prior cardiovascular disease (discussed below). All other studies specifically excluded participants with prior cardiovascular diseases or symptoms. The third analysis was on findings of ENDS use of 1 year or longer. Two studies reported no modification on HR [35, 44], and one study reported no significant changes in either BP or HR [51].

Two studies that measured BP after 1 year of ENDS substitution found clinically relevant reductions in participants with hypertension. Farsalinos et al. [35] conducted a sub-group analysis of 66 participants with elevated BP at baseline. Their average baseline systolic BP 141.2 dropped to 132.4 (*p* < 0.001), plus they had a significant but small decrease in diastolic BP. A clinically significant reduction in BP was observed by Polosa et al. [44] in a cohort of 43 participants with hypertension. After 1 year, participants using ENDS exclusively experienced a reduction in systolic BP from 140 to 130; dual users also had significant reductions.

The final analysis evaluated the overall confidence in the evidence with the GRADE rating. It was assessed as low to very low. Twelve of the clinical trials were lowered from high certainly to low due to the high risk of bias. Eight clinical trials were lowered from high to very low for high risk of bias plus either imprecision for excessive ENDS exposure or indirectness for testing with discontinued early ENDS models. The one observational study was lowered from low to very low for some concerns of bias. For the four studies at the moderate rating, three had no significant findings and one study had significant findings only for a sub-group. No studies were at low risk of bias; therefore, none were eligible for a higher rating. See Online Resources Document 12, GRADE rating.

## Discussion

We found discrepant results regarding the effects of ENDS substitution on cardiovascular outcomes. The findings had no indications that ENDS use is more harmful than smoking. In 14 studies, ENDS substitution induced no significant changes in HR. In the five studies that recorded in a slight increase in HR, one study found that the increase was lower with ENDS compared to tobacco smoking, and in two studies, the increase was not significantly different between them. In 15 studies, ENDS use had no significant effect on BP, and a beneficial effect in three. Of the 55 tests extracted from the studies, 36 tests, just under two-thirds did not show significant changes in cardiovascular function with ENDS use. The GRADE quality of evidence is low to very low.

### Study quality issues: methodology

We identified substantive problems with the research designs of many studies.

Blinding was not clear or absent in many studies. Although blinding participants, testers (clinical testers), and assessors (data analysts) is the standard, blinding participants is not always possible in ENDS clinical research. Participants certainly see the difference between ENDS and cigarettes or the lack of vapor with sham vaping. Nevertheless, nicotine levels can be masked. In the 14 RCTs, seven did not specify the blinding of the treaters and nine did not report blinding of assessors.

Ascertainment bias was common in the studies. It can be introduced when participants in a clinical trial differ from the target population [58]. For gender, males were frequently dominant in the participant populations; three studies were conducted with only male participants, and nine studies had a substantially higher percentage of male participants. Conversely, one study had all female participants and three had a substantially higher percentage of female participants. Patterns of cigarette use by participants varied. Three studies included participants smoking less than 10 cigarettes a day, and fourteen studies included participants smoking more than 20 cigarettes a day (heavy smoking). Only one study included smoking history in their analysis, and half the studies did not report the smoking history of the participants. For studies on cardiovascular functioning, the age of participants is critical; the age for CVD risk is ≥ 40 years [59]. Four studies included participants exclusively over age 40; nine studies included participants over 40 but with an average age under 40; and six studies had no participants over age 40. The divergent factors of gender, age, and patterns of cigarette use limit the generalizability of the findings.

Different ENDS models are an issue with evidence from early studies. ENDS models are continuously evolving, so findings based on the first devices may not be applicable to current ones [60]. Five studies were conducted with the now discontinued “cig-a-like” models; they complicate the generalizability of study findings [61].

Finally, bias occurs when acute studies are conducted with vaping protocols that do not replicate how individuals use ENDS in real life [62]. The standard laboratory exposure is 15 puffs in 10 min based on the widely accepted Penn State Electronic Cigarette Dependence Index [63]. An excessive exposure protocol was conducted in three studies.

### Study quality issues: reporting

Omissions and errors in reporting in the studies led to serious concerns for potential bias (see Online Document 11, Bias table).

Changes from the protocol of a study should be disclosed. Substantial deviations from protocol may be an indicator of data dredging [64]. Thirteen studies either did not report a protocol or the protocol was not published, thereby limiting an examination for missing data [65, 66] or the identification of changes in the designation of primary and secondary outcomes [67, 68]. Of the 12 studies with a published protocol, nine had deviations that were not acknowledged in the published study; two of which had substantial deviations.

Reporting errors occur when there are discrepancies within the article between the data reported in the abstract, discussion, tables, figures, or conclusions [24]. Ten publications had data discrepancies. These errors distorted the presentation of data, including in the conclusions.

Bias may occur from how findings are reported. In nine studies, their discussions and conclusions were presented with spin bias. This occurs when authors, intentionally or unintentionally, emphasize *p* values [69] or secondary endpoints without clinical relevance [70, 71]. Some studies failed to discuss all their data or framed non-significant tests as substantive evidence. Furthermore, authors engaged in one-sided reference bias or “all’s well” literature bias by citing only studies that supported their results while omitting those opposing them [72].

Finally, studies that fail to find any significant findings may push researchers into spin bias with speculations, forgetting that non-significant results are important research findings [73]. In our review, demonstrating that ENDS have no significant difference in cardiovascular effects compared to TC is highly relevant.

### Comparison with other systematic reviews

Other systematic reviews have included data from study designs from in vitro, animal, biomarkers, and cross-sectional surveys. We excluded these study designs, because direct evidence for human health effects cannot be obtained from them. First of all, cross-sectional studies cannot prove causation, because the temporal sequence of ENDS use and disease outcomes cannot be established. In vitro studies “still do not fully represent complex in vivo systems and may not directly translate to adverse effects relevant to disease outcomes” [60; see also 74]. Plus, the predictive capacity of in vitro testing is unknown [60]. In animal tests on ENDS (almost all with rats), exposures are performed with intra-tracheally or nasally administered liquids, or whole-body aerosol exposure, protocols that do not reflect human exposure levels [75]. Finally, biomarkers are surrogate outcomes [76]. Their role is prominent in screening but remains limited in indicating disease [77]. Furthermore, the predictive value of biomarkers for tobacco-related diseases has yet to be verified [78, 79].

Bearing in mind the critical limitations of these study designs, we compared our findings with the five recent systematic reviews on cardiovascular outcomes.

Martinez-Morata, Sanchez, Shimbo and Navas-Acien [6] with 13 studies, concluded that ENDS may cause elevations in BP. Their evidence included ENDS use by people who do not smoke, so do not reflect substitution effects. No quality appraisal was performed.

Kennedy, van Schalkwyk, McKee and Pisinger [80] analysed 38 studies, including 6 animal and 8 in vitro studies. The authors excluded studies which they deemed to have a conflict of interest. They concluded that 90% of the studies found potentially harmful effects on the cardiovascular system. Yet, 6 of 11 studies at moderate–high risk of bias had conclusions that were supportive of ENDS use, because their negative effects were lower than TC. Their assessments of issues with the randomization and blinding in studies were very similar to ours.

The Goniewicz et al.’s [81] meta-analysis was calculated based on three cross-sectional population studies on myocardial infarction, coronary heart disease, and stroke. They concluded that there were no significant differences in cardiovascular outcomes between former smokers who transitioned to ENDS compared to current exclusive smokers.

Two systematic reviews conducted a meta-analysis of BP and HR outcomes. Garcia et al. [62] examined 19 studies and presented conclusions similar to ours that acute ENDS exposure increases HR and BP less than TC. Similar findings were reported by Skotsimara et al. [82] with a meta-analysis of 14 studies, although their conclusion focused on negative effects. Both reviews contained some concerns of imprecision, did not identify clinically relevant results, and neither conducted a quality assessment.

### Future research

Initial findings in this systematic review show promise for harm reduction with ENDS substitution for smoking for persons with hypertension. A reduction of 10 mmHg in SBP significantly reduces the risk for major cardiovascular events with a relative risk of 0.80 (95% CI 0.77–0.83) [83]. Two studies, *N* = 109, rated at some concerns of bias found a mean reduction ~ 10 mmHg for participants with hypertension who switched to ENDS for 1 year. This potential beneficial effect deserves further study with clinical testing and medical record cohort studies, preferably longer term.

Based on this review, we offer these recommendations for future ENDS research:

- Reconsider the value of acute testing. “Acute studies of effects of ENDS in humans may not reflect long term effects” [84]. Nonetheless, for RCTs conducting acute ENDS exposure, administer a validated puff protocol such as the Pennsylvania University standard [63].
- Carry out blinding procedures where feasible. A good example is the study by Veldheer et al. [49] for its processes for nicotine content labeling and participant identification.
- Account for the ascertainment bias factors specific to smoking: smoking history which includes quit attempts and patterns of use which includes dual use. These participant profiles should be considered for both recruiting and in analyses.
- Eliminate reporting errors. Data discrepancies in a study article should be rooted out by the authors, or failing that, peer reviewers.
- Clearly differentiate in the Discussion and conclusions between statistical significance and clinical relevance, because a numerical change in a clinical test measurement may not indicate a change in health status. For example, a change in heart rate from a baseline reading of 65.7 (± 7.7) to 71.6 (± 8.1) 5 min after ENDS use is not of clinical relevance but generates a *p* < 0.05 [study 39].
- And a respectful reminder: publishing a protocol is a must for transparency and confidence.

### Limitations

The studies retrieved for the review have many limitations. The large majority, 80%, were rated at high risk of bias, and no studies were at low risk. Nearly half of the studies were conducted with small sample sizes. These and other issues resulted in the GRADE assessment of the evidence to be at low to very low certainty. In addition, acute effects contribute little to understanding health outcomes, nor do significant *p* values indicate clinically relevant outcomes. Finally, there were few long-term studies.

For the systematic review, our analysis method, vote counting of direction of effect, is limited. It does not provide data on the magnitude of the effect size, nor of its clinical relevance. This method does not represent the relative sizes of the studies, and many of the studies had a small number of participants [22].

To achieve the highest quality, this review is PRISMA 2020 compliant [19] and meets all AMSTAR2 standards [85]. Unfortunately, the most rigorous methodology cannot overcome the limitations caused by the poor quality of the available studies.

## Conclusions

The large majority of studies, nearly two-thirds, found no significant changes in heart rate or blood pressure with ENDS substitution for smoking. Where ENDS use increased the heart rate, in most cases the increase was lower compared to smoking. One-year use of ENDS resulted in improvements in hypertension for 109 participants in two studies. Our conclusion is that ENDS substitution is not more harmful to health than continued smoking and may have some limited benefits. Our confidence in these conclusions is low to very low.

Furthermore, based on our quality and bias assessments, the current research needs to be read with a jaundiced eye. For future studies, we urge researchers to pay close attention to their research designs and reporting of data. More quality evidence is certainly needed to inform if ENDS substitution is a worthwhile option for harm reduction for persons who smoke.

## Supplementary Information

Below is the link to the electronic supplementary material.

Supplementary file1 (DOCX 460 kb)

## Data Availability

All data generated or analysed during this review and included in the published article and Online Resources.

## Declarations

### Data repository

Data extraction forms, the JBI forms, Bias assessment forms, and Data discrepancy reports are available at Zenodo https://zenodo.org/record/4835883#.YPnMqECxUuU.
