# Supplementary material for: Clinical testing of the cardiovascular effects of e-cigarette substitution for smoking: a living systematic review
Source: Intern Emerg Med. 2023 Jan 7;18(3):917–28. doi: 10.1007/s11739-022-03161-z (PMC10081981; doi:10.1007/s11739-022-03161-z)
Supplement: Supplementary file 1 — Supplementary file1 (DOCX 460 kb) [file 11739_2022_3161_MOESM1_ESM.docx]

**Online Resources**

**Clinical testing of the cardiovascular effects of e-cigarette substitution for smoking: a living systematic review**

**Internal and Emergency Medicine**

Giusy La Rosa^a^; Robin Vernooij^a,b^; Maria Qureshi^a,c^; Riccardo Polosa^a,d^; Renee O'Leary^d*^

1. *Department of Clinical and Experimental Medicine, University of Catania, Catania, Italy.*
2. *Current position: Department of Nephrology and Hypertension, University Medical Center Utrecht, Utrecht, the Netherlands; Julius Center for Health Sciences and Primary Care, University Medical Center Utrecht, Utrecht University, Utrecht, the Netherlands.*
3. *Current position: Center for the Acceleration of Harm Reduction, University of Catania, Catania, Italy.*
4. *Center for the Acceleration of Harm Reduction, University of Catania, Catania, Italy.*

**Corresponding Author Renée O’Leary renee.oleary@eclatrbc.it*

Table of Contents

Document 1 PRISMA checklist

Document 2 Search syntax

Document 3 Grey literature searches

Document 4 Data extraction form

Document 5 Bias report form

Document 6 Bias rating rubric

Document 7 Protocol deviations report

Document 8 Data discrepancies report

Document 9 Excluded studies

Document 10 Study evidence table

Document 11 Study biases

Document 12 GRADE analysis

**
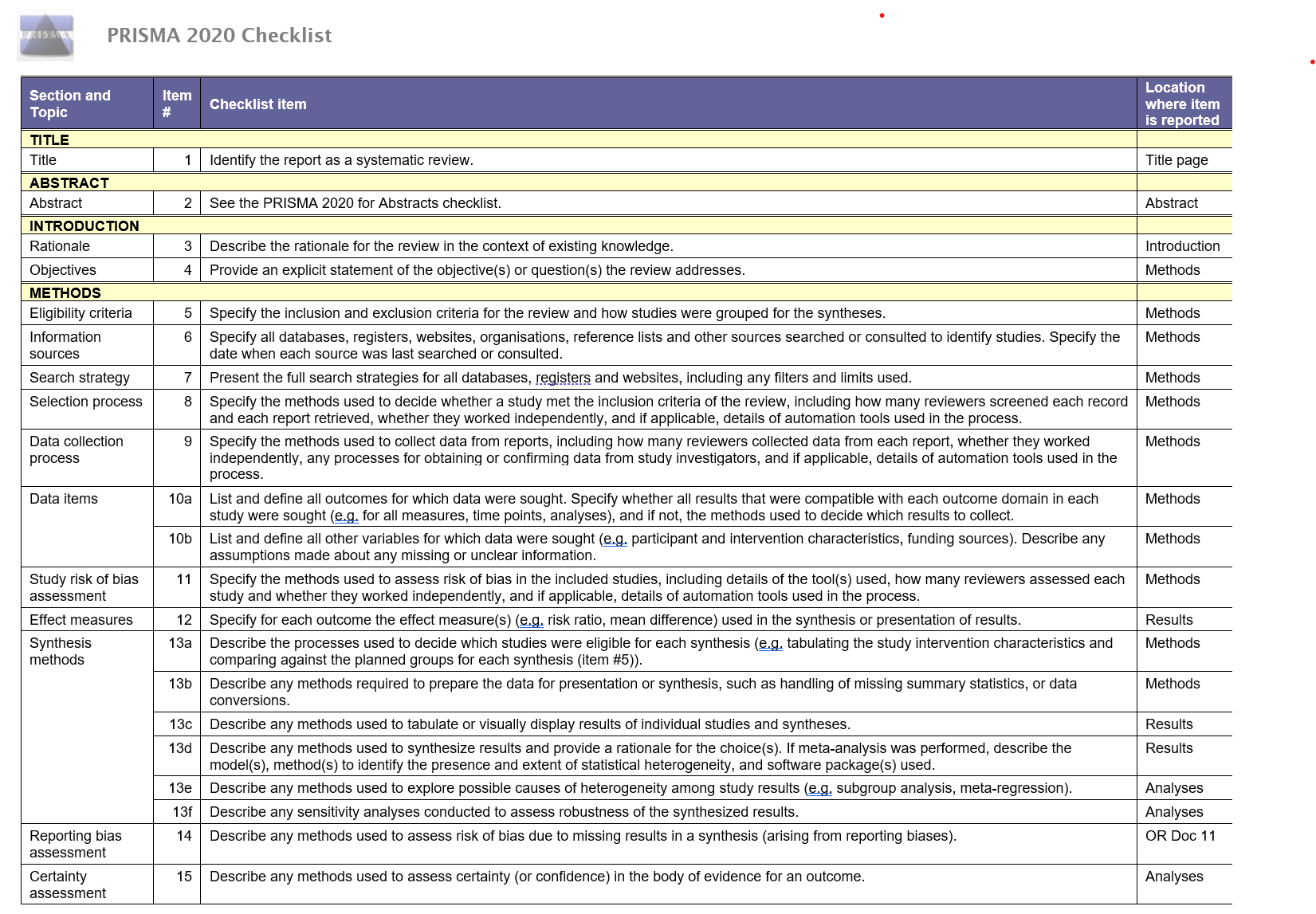
**

**
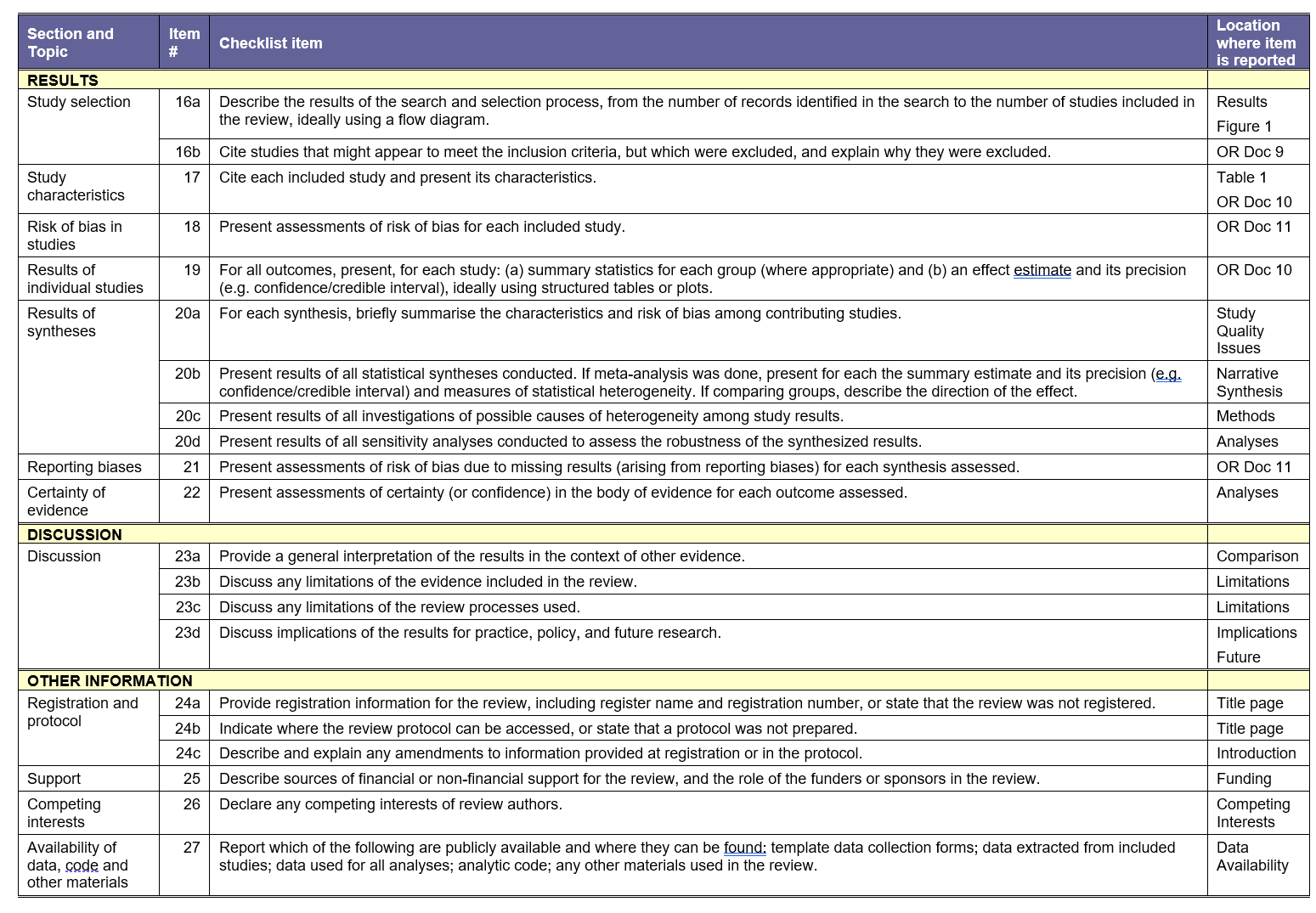
**

**Online Resource Document 2 Search syntax**

((“electronic cigarette” [title/abstract] OR e-cigarette [title/abstract]) AND (cardiovascular OR heart OR circulatory OR arterial OR stroke))


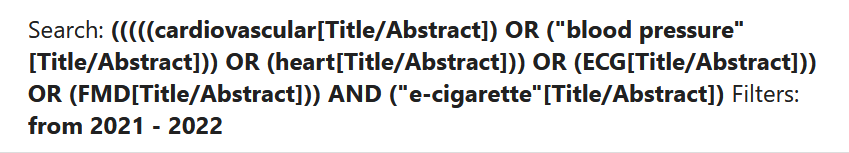


**Online Document 3 Grey literature searches**

| American Heart Organisation | <https://www.heart.org/> |
| --- | --- |
| European Society of Cardiology | <https://www.escardio.org/> |
| American College of Cardiology | <https://www.acc.org/#sort=%40commonsortdate%20descending> |
| Indian Association of Clinical Cardiologist | <https://www.accindia.org/> |
| British Cardiovascular Society | <https://www.britishcardiovascularsociety.org/> |
| British Junior Cardiologist Association | <https://bjca.tv/> |
| Canadian Cardiovascular Society | <https://ccs.ca/> |
| Cardiac Society of Australia & New Zealand | <https://www.csanz.edu.au/> |
| Irish Cardiac Society | <https://www.irishcardiacsociety.com/pages/default.asp> |
| Emirates Cardiac Society | <https://ecsociety.com/> |
| Caribbean Cardiac Society | <https://cardiac.memberclicks.net/> |
| Scottish Cardiac Society | <https://scottishcardiac.org/> |
| Cardiac Society of Nepal | <http://www.csn.org.np/> |
| Pakistan Cardiac Society | <http://www.pcs.org.pk/> |
| Adult Congenital Heart Association | <https://www.achaheart.org/> |
| Gulf Heart Association | <https://gulfheart.org/> |
| Philippines Heart Association | <https://www.philheart.org/> |
| Italian Federation of Cardiologists | <https://www.federcardio.it/en> |
| Singapore Cardiac Society | <https://www.singaporecardiac.org/> |
| Heart and Stroke Foundation of Canada | <https://www.heartandstroke.ca/> |
| Heart Rhythm Society | <https://www.hrsonline.org/> |
| Atrial Fibrillation Association | <https://www.heartrhythmalliance.org/afa/uk/> |
| World Heart Federation | <https://www.world-heart-federation.org/> |
| British Heart Foundation | <https://www.bhf.org.uk/> |
| Royal College of Physicians | <https://www.rcplondon.ac.uk/> |
| American College of Chest Physicians | <https://www.chestnet.org/> |
| Cardiac Risk in the Young | <https://www.c-r-y.org.uk/> |
| Thrombosis UK | <https://thrombosisuk.org/> |
| Stroke Association | <https://www.stroke.org.uk/> |
| American Stroke Association | <https://www.stroke.org/> |
| World Stroke Organisation | <https://www.world-stroke.org/> |
| European Stroke Organisation | <https://eso-stroke.org/> |
| Singapore National Stroke Organisation | <https://www.snsa.org.sg/> |
| Stroke Foundation | <https://strokefoundation.org.au/> |
| Indian Stroke Association | <http://www.stroke-india.org/> |
| Pacific Stroke Organisation | <https://pacificstrokeassociation.org/> |
| Stroke Society of Australasia | <https://www.strokesociety.com.au/> |
| Nordic Stroke Society | <https://nordicstroke.org/> |
| Saudi Stroke Society | <https://stroke.org.sa/> |
| Pakistan Stroke Society | <http://pakstroke.com/> |
| Korean Stroke Society | <https://www.stroke.or.kr:4454/eng/> |

**Online Resource 4 Data extraction form**

Data Extraction Form

Reviewer: Date: Verified on:

Title:

Year:

Journal: DOI: PMID:

1^st^ Author:

Affiliations:

COI Declaration:

2^nd^ Author

Affiliations:

COI Declarations:

[all additional authors]

Corresponding author and email:

Additional/supplementary files [identify or NONE]

Published protocol or trial registry [identify or NONE]

Funder:

Research question (or goal if no stated question)

Study design:

Location (Country):

Setting:

Participants:

Age

Sex

Exclusion criteria

Smoking status/history/current use

Other tobacco products reported

ENDS history

Disease status

Other descriptors (economic status, etc.)

Recruitment and Compensation

Randomization/assignment process

Statistical power

Intervention

ENDS device and liquid

ENDS training

Frequency of use

Duration of exposure

Concurrent use of tobacco

Follow-up periods

Drop-outs

Fidelity including relapse to cigarette use during study

Tests/data

Data collection methods

Named tests

Test not eligible for data extraction

Testing protocols including pretest abstinence period

Observational data (disease symptoms)

Self-report data

Verification of tobacco abstinence

Tests results/data [for each follow-up period]

Tests

Observational data

Self-report data

Dual user and exclusive users reported separately or combined

Secondary outcomes (tobacco cessation, ENDS cessation, tobacco relapse)

Statistical tests

Statistical analyses

Limitations identified by authors

Recommendations for future research

Conclusions(s) quotations (with page number)

Reviewer Comments:

**Online Resource 5 Bias report form**

Biases Reporting

Reviewer date verification

Author (year)

Reporting Biases

Spin Bias

# Data-dredging bias

# Hypothetical bias

## All’s well literature bias

## Ascertainment bias

## Biases of rhetoric

## Compliance bias

## Confirmation bias

## Detection bias

## Hot stuff bias

## Industry Sponsorship Bias

## Misclassification bias

## One-sided reference bias

## Partial reference bias

## Performance bias

## Popularity bias

## Prevalence-incidence (Neyman) bias

## Selection bias

## Substitution game bias

## Volunteer bias

## Wrong sample size bias

**Online Resource 6 Bias rating rubric**

| **Rating** | **JBI (participant blinding excluded TC vs ENDS)** | **Internal Validity** |
| --- | --- | --- |
| LOW | All yes | none |
| SOME CONCERNS | All yes | 1-2 |
| SOME CONCERNS | One no | 0-1 |
| HIGH | More than one no | any |
| HIGH | All yes | 3 or more |

**Online Resource 7 Protocol deviations checklist**

Protocol/Registry Study Deviations Report

Reviewer Date Verified on

Author (year)

Status (select):

No protocol/trial registry reported in study.

Protocol/trial registry reported in study, not published.

Protocol/trial registry published.

Deviations from protocol/trial registry reported by study authors:

Deviations from published protocol/trial registry observed by reviewer:

**Online Resource 8 Data discrepancies report**

Data Discrepancy Form

Reviewer: Date: Verified on:

Author (year):

Indicate status:

No discrepancies observed.

Discrepancies in data between the abstract and the study text:

Discrepancies within the text (compare all references in the text to the data):

Discrepancies between the text and figure:

Discrepancies between the text and table:

Discrepancies in number of participants:

Study corresponding author contacted on date and text of email:

(If no reply): Other authors and cc of journal editor on date and text of email:

Review paper: flagged in study table, noted in QA.

**Online Resources 9 Excluded studies**

Not an included study design

Alzahrani, T., I. Pena, N. Temesgen and S. A. Glantz (2018). "Association Between Electronic Cigarette Use and Myocardial Infarction." American Journal of Preventive Medicine **55**(4): 455-461.

Boas, Z., P. Gupta, R. S. Moheimani, M. Bhetraratana, F. Yin, K. M. Peters, J. Gornbein, J. A. Araujo, J. Czernin and H. R. Middlekauff (2017). "Activation of the “Splenocardiac Axis” by electronic and tobacco cigarettes in otherwise healthy young adults." Physiological Reports **5**(17).

Ikonomidis, I., D. Vlastos, K. Kourea, G. Kostelli, M. Varoudi, G. Pavlidis, P. Efentakis, H. Triantafyllidi, J. Parissis, I. Andreadou, E. Iliodromitis and J. Lekakis (2018). "Electronic Cigarette Smoking Increases Arterial Stiffness and Oxidative Stress to a Lesser Extent Than a Single Conventional Cigarette: An Acute and Chronic Study." Circulation **137**(3): 303-306.

Kim, C. Y., Y. J. Paek, H. G. Seo, Y. S. Cheong, C. M. Lee, S. M. Park, D. W. Park and K. Lee (2020). "Dual use of electronic and conventional cigarettes is associated with higher cardiovascular risk factors in Korean men." Scientific Reports **10**(1).

Vlachopoulos, C., N. Ioakeimidis, M. Abdelrasoul, D. Terentes-Printzios, C. Georgakopoulos, P. Pietri, C. Stefanadis and D. Tousoulis (2016). "Electronic Cigarette Smoking Increases Aortic Stiffness and Blood Pressure in Young Smokers." J Am Coll Cardiol **67**(23): 2802-2803.

No comparator to smoking

Antoniewicz, L., J. A. Bosson, J. Kuhl, S. M. Abdel-Halim, A. Kiessling, F. Mobarrez and M. Lundbäck (2016). "Electronic cigarettes increase endothelial progenitor cells in the blood of healthy volunteers." Atherosclerosis **255**: 179-185.

Antoniewicz, L., A. Brynedal, L. Hedman, M. Lundbäck and J. A. Bosson (2019). "Acute Effects of Electronic Cigarette Inhalation on the Vasculature and the Conducting Airways." Cardiovascular Toxicology **19**(5): 441-450.

Arefalk, G., K. Hambraeus, L. Lind, K. Michaelsson, B. Lindahl and J. Sundstrom (2014). "Discontinuation of smokeless tobacco and mortality risk after myocardial infarction." Circulation **130**(4): 325-332.

Caporale, A., M. C. Langham, W. Guo, A. Johncola, S. Chatterjee and F. W. Wehrli (2019). "Acute Effects of Electronic Cigarette Aerosol Inhalation on Vascular Function Detected at Quantitative MRI." Radiology **293**(1): 97-106.

Chaumont, M., B. De Becker, W. Zaher, A. Culié, G. Deprez, C. Mélot, F. Reyé, P. Van Antwerpen, C. Delporte, N. Debbas, K. Z. Boudjeltia and P. Van De Borne (2018). "Differential Effects of E-Cigarette on Microvascular Endothelial Function, Arterial Stiffness and Oxidative Stress: A Randomized Crossover Trial." Scientific Reports **8**(1).

Chaumont, M., V. Tagliatti, E. M. Channan, J. M. Colet, A. Bernard, S. Morra, G. Deprez, A. van Muylem, N. Debbas, T. Schaefer, V. Faoro and P. van de Borne (2020). "Short halt in vaping modifies cardiorespiratory parameters and urine metabolome: A randomized trial." American Journal of Physiology - Lung Cellular and Molecular Physiology **318**(2): L331-L344.

Cossio, R., Z. A. Cerra and H. Tanaka (2020). "Vascular effects of a single bout of electronic cigarette use." Clinical and Experimental Pharmacology and Physiology **47**(1): 3-6.

Goniewicz, M. L., M. Gawron, D. M. Smith, M. Peng, P. Jacob, 3rd and N. L. Benowitz (2017). "Exposure to Nicotine and Selected Toxicants in Cigarette Smokers Who Switched to Electronic Cigarettes: A Longitudinal Within-Subjects Observational Study." Nicotine Tob Res **19**(2): 160-167.

Gonzalez, J. E. and W. H. Cooke (2021). "Acute effects of electronic cigarettes on arterial pressure and peripheral sympathetic activity in young nonsmokers." Am J Physiol Heart Circ Physiol **320**(1): H248-h255.

Hajek, P., A. Phillips-Waller, D. Przulj, F. Pesola, K. Myers Smith, N. Bisal, J. Li, S. Parrott, P. Sasieni, L. Dawkins, L. Ross, M. Goniewicz, Q. Wu and H. J. McRobbie (2019). "A Randomized Trial of E-Cigarettes versus Nicotine-Replacement Therapy." N Engl J Med **380**(7): 629-637.

Haptonstall, K. P., Choroomi, Y., Moheimani, R., Nguyen, K., Tran, E., Lakhani, K., Ruedisueli, I., Gornbein, J., & Middlekauff, H. R. (2020). Differential effects of tobacco cigarettes and electronic cigarettes on endothelial function in healthy young people. *American Journal of Physiology - Heart and Circulatory Physiology, 319*(3), H547-H556. https://doi.org/10.1152/ajpheart.00307.2020

Hiler, M., N. Karaoghlanian, S. Talih, S. Maloney, A. Breland, A. Shihadeh and T. Eissenberg (2020). "Effects of electronic cigarette heating coil resistance and liquid nicotine concentration on user nicotine delivery, heart rate, subjective effects, puff topography, and liquid consumption." Experimental and Clinical Psychopharmacology **28**(5): 527-539.

Hughes, J. R., E. N. Peters, P. W. Callas, C. Peasley-Miklus, E. Oga, J. F. Etter and N. Morley (2020). "Withdrawal Symptoms from E-Cigarette Abstinence among Adult Never-Smokers: A Pilot Experimental Study." Nicotine and Tobacco Research **22**(5): 740-746.

Hughes, J. R., E. N. Peters, P. W. Callas, C. Peasley-Miklus, E. Oga, J. F. Etter and N. Morley (2020). "Withdrawal Symptoms from E-Cigarette Abstinence among Former Smokers: A Pre-Post Clinical Trial." Nicotine and Tobacco Research **22**(5): 734-739.

Kinoshita, M., R. M. Herges, D. O. Hodge, L. Friedman, N. M. Ammash, C. J. Bruce, V. Somers, J. F. Malouf, J. Askelin, J. A. Gilles, B. J. Gersh and P. A. Friedman (2009). "Role of smoking in the recurrence of atrial arrhythmias after cardioversion." Am J Cardiol **104**(5): 678-682.

Lee, M. S., V. W. Rees, P. Koutrakis, J. M. Wolfson, Y. S. Son, J. Lawrence and D. C. Christiani (2019). "Cardiac Autonomic Effects of Secondhand Exposure to Nicotine from Electronic Cigarettes: An Exploratory Study." Environ Epidemiol **3**(1).

McClelland, M. L., C. S. Sesoko, D. A. MacDonald and L. M. Davis (2020). "Effects on vital signs after twenty minutes of vaping compared to people exposed to second-hand vapor." Advances in respiratory medicine **88**(6): 504-514.

Moheimani, R. S., M. Bhetraratana, K. M. Peters, B. K. Yang, F. Yin, J. Gornbein, J. A. Araujo and H. R. Middlekauff (2017). "Sympathomimetic effects of acute e-cigarette use: Role of nicotine and non-nicotine constituents." Journal of the American Heart Association **6**(9).

Nadruz, W., Jr., B. Claggett, A. Goncalves, G. Querejeta-Roca, M. M. Fernandes-Silva, A. M. Shah, S. Cheng, H. Tanaka, G. Heiss, D. W. Kitzman and S. D. Solomon (2016). "Smoking and Cardiac Structure and Function in the Elderly: The ARIC Study (Atherosclerosis Risk in Communities)." Circ Cardiovasc Imaging **9**(9): e004950.

Polosa, R., F. Cibella, P. Caponnetto, M. Maglia, U. Prosperini, C. Russo and D. Tashkin (2017). "Health impact of E-cigarettes: A prospective 3.5-year study of regular daily users who have never smoked." Scientific Reports **7**(1).

Pywell, M. J., M. Wordsworth, R. M. Kwasnicki, P. Chadha, S. Hettiaratchy and T. Halsey (2018). "The Effect of Electronic Cigarettes on Hand Microcirculation." J Hand Surg Am **43**(5): 432-438.

Rüther, T., D. Hagedorn, K. Schiela, T. Schettgen, H. Osiander-Fuchs and W. Schober (2018). "Nicotine delivery efficiency of first- and second-generation e-cigarettes and its impact on relief of craving during the acute phase of use." International Journal of Hygiene and Environmental Health **221**(2): 191-198.

Spindle, T. R., M. M. Hiler, A. B. Breland, N. V. Karaoghlanian, A. L. Shihadeh and T. Eissenberg (2017). "The Influence of a Mouthpiece-Based Topography Measurement Device on Electronic Cigarette User's Plasma Nicotine Concentration, Heart Rate, and Subjective Effects Under Directed and Ad Libitum Use Conditions." Nicotine & tobacco research : official journal of the Society for Research on Nicotine and Tobacco **19**(4): 469-476.

St.Helen, G., C. Havel, D. A. Dempsey, P. Jacob, III and N. L. Benowitz (2016). "Nicotine delivery, retention and pharmacokinetics from various electronic cigarettes." Addiction **111**(3): 535-544.

Wallenfeldt, K., J. Hulthe, L. Bokemark, J. Wikstrand and B. Fagerberg (2001). "Carotid and femoral atherosclerosis, cardiovascular risk factors and C-reactive protein in relation to smokeless tobacco use or smoking in 58-year-old men." J Intern Med **250**(6): 492-501.

Yatsuya, H., A. R. Folsom and A. Investigators (2010). "Risk of incident cardiovascular disease among users of smokeless tobacco in the Atherosclerosis Risk in Communities (ARIC) study." Am J Epidemiol **172**(5): 600-605.

No data on cardiovascular functions or diseases

Bullen, C., C. Howe, M. Laugesen, H. McRobbie, V. Parag, J. Williman and N. Walker (2013). "Electronic cigarettes for smoking cessation: a randomised controlled trial." Lancet **382**(9905): 1629-1637.

Czogała, J., M. Cholewiński, A. Kutek and W. Zielińska-Danch (2012). "[Evaluation of changes in hemodynamic parameters after the use of electronic nicotine delivery systems among regular cigarette smokers]." Przegla̧d lekarski **69**(10): 841-845.

Flouris, A. D., K. P. Poulianiti, M. S. Chorti, A. Z. Jamurtas, D. Kouretas, E. O. Owolabi, M. N. Tzatzarakis, A. M. Tsatsakis and Y. Koutedakis (2012). "Acute effects of electronic and tobacco cigarette smoking on complete blood count." Food Chem Toxicol **50**(10): 3600-3603.

Hébert-Losier, A., K. B. Filion, S. B. Windle and M. J. Eisenberg (2020). "A Randomized Controlled Trial Evaluating the Efficacy of E-Cigarette Use for Smoking Cessation in the General Population: E3 Trial Design." CJC Open **2**(3): 168-175.

Hecht, S. S., S. G. Carmella, D. Kotandeniya, M. E. Pillsbury, M. Chen, B. W. Ransom, R. I. Vogel, E. Thompson, S. E. Murphy and D. K. Hatsukami (2015). "Evaluation of toxicant and carcinogen metabolites in the urine of e-cigarette users versus cigarette smokers." Nicotine Tob Res **17**(6): 704-709.

Manzoli, L., C. La Vecchia, M. E. Flacco, L. Capasso, V. Simonetti, S. Boccia, A. Di Baldassarre, P. Villari, A. Mezzetti and G. Cicolini (2013). "Multicentric cohort study on the long-term efficacy and safety of electronic cigarettes: Study design and methodology." BMC Public Health **13**(1).

Wagener, T. L., E. L. Floyd, I. Stepanov, L. M. Driskill, S. G. Frank, E. Meier, E. L. Leavens, A. P. Tackett, N. Molina and L. Queimado (2017). "Have combustible cigarettes met their match? The nicotine delivery profiles and harmful constituent exposures of second-generation and third-generation electronic cigarette users." Tobacco Control **26**(e1): e23-e28.

No substitution of ENDS for smoking

Arastoo, S., Haptonstall, K. P., Choroomi, Y., Moheimani, R., Nguyen, K., Tran, E., Gornbein, J., & Middlekauff, H. R. (2020). Acute and chronic sympathomimetic effects of e-cigarette and tobacco cigarette smoking: Role of nicotine and non-nicotine constituents. *American Journal of Physiology - Heart and Circulatory Physiology, 319*(2), H262-H270. https://doi.org/10.1152/ajpheart.00192.2020

Benowitz, N. L., St. Helen, G., Nardone, N., Addo, N., Zhang, J., Harvanko, A. M., Calfee, C. S., & Jacob, P. (2020). Twenty‐Four‐Hour Cardiovascular Effects of Electronic Cigarettes Compared With Cigarette Smoking in Dual Users. *Journal of the American Heart Association, 9*(23). https://doi.org/10.1161/jaha.120.017317

Farsalinos, K. E., Tsiapras, D., Kyrzopoulos, S., Savvopoulou, M., & Voudris, V. (2014). Acute effects of using an electronic nicotine-delivery device (electronic cigarette) on myocardial function: comparison with the effects of regular cigarettes. *BMC Cardiovasc Disord, 14*, 78. https://doi.org/10.1186/1471-2261-14-78

Fetterman, J. L., Keith, R. J., Palmisano, J. N., McGlasson, K. L., Weisbrod, R. M., Majid, S., Bastin, R., Stathos, M. M., Stokes, A. C., Robertson, R. M., Bhatnagar, A., & Hamburg, N. M. (2020). Alterations in Vascular Function Associated With the Use of Combustible and Electronic Cigarettes. *Journal of the American Heart Association, 9*(9), e014570. https://doi.org/10.1161/JAHA.119.014570

Ip, M., Diamantakos, E., Haptonstall, K., Choroomi, Y., Moheimani, R. S., Nguyen, K. H., Tran, E., Gornbein, J., & Middlekauff, H. R. (2020). Tobacco and electronic cigarettes adversely impact ECG indexes of ventricular repolarization: Implication for sudden death risk. *American Journal of Physiology - Heart and Circulatory Physiology, 318*(5), H1176-H1184. https://doi.org/10.1152/AJPHEART.00738.2019

Manzoli, L., Flacco, M. E., Fiore, M., La Vecchia, C., Marzuillo, C., Gualano, M. R., Liguori, G., Cicolini, G., Capasso, L., D'Amario, C., Boccia, S., Siliquini, R., Ricciardi, W., & Villari, P. (2015). Electronic cigarettes efficacy and safety at 12 months: Cohort study. *PLoS ONE, 10*(6), Article e0129443. https://doi.org/10.1371/journal.pone.0129443

Osibogun, O., Bursac, Z., McKee, M., Li, T., & Maziak, W. (2020). Cessation outcomes in adult dual users of e-cigarettes and cigarettes: the Population Assessment of Tobacco and Health cohort study, USA, 2013–2016. *International Journal of Public Health, 65*(6), 923-936. https://doi.org/10.1007/s00038-020-01436-w

Other – see notes

MacLean, R. R., R. Gueorguieva, E. E. DeVito, M. R. Peltier, S. Parida and M. Sofuoglu (2020). "The Effects of Inhaled Flavors on Intravenous Nicotine." Experimental and Clinical Psychopharmacology. Intravenous administration, not vaping.

Walker, N., Parag, V., Verbiest, M., Laking, G., Laugesen, M., & Bullen, C. (2020). Nicotine patches used in combination with e-cigarettes (with and without nicotine) for smoking cessation: a pragmatic, randomised trial. *The Lancet Respiratory Medicine, 8*(1), 54-64. https://doi.org/10.1016/S2213-2600(19)30269-3 ENDS combined with nicotine replacement therapy patch.

**Online Resource 10 Study evidence table**

| **Author Year Country Funder** | **Research design**  **Location**  **Bias Rating** | **Participants** | **Intervention** | **Frequency of testing Tests** | **Results BL to final test/end of study** |
| --- | --- | --- | --- | --- | --- |
| **Acute** |  |  |  |  |  |
| Biondi-Zoccai, 2019,  Italy  University funder | RCT  cross-over  Location: NR  High risk of bias | N=20  Gender: 6 male, 14 female  Age: 35 (13)  Smoking history: NR.  TC: 11-30/day | Exposures: heat-not-burn cigarette, ENDS, TC.  9 puffs  6 mg/ml nicotine | Post-exposure time NR  SBP  DBP  MBP  FMD | **Within subject**  **SBP:** ENDS 121.7 (6.5) to 130.6 (6.5) *p*< 0.001  TC 121.5 (8.3) to 132.4 (6.2) *p*< 0.001  **DBP:** ENDS 72.2 (4.4) to 78.0 (4.8) *p*< 0.001  TC 73.3 (4.8) to 80.2 (5.2) *p*< 0.001  **MBP:** ENDS 88.7(3.6) to 95.5(3.6) *p* <0.001  TC 89.4 (4.7) to 97.6 (3.4) *p*< 0.001  **FMD:** ENDS 6.14% (3.17) to 3.72% (3.14) *p* <0.001  TC 6.20% (3.26) to 2.40% (1.89) *p*< 0.001  **ENDS vs. TC**: NS |
| Carnevale 2016  Mastrangli 2018  Italy  University funder | Quasi-experimental  Location: NR  Some bias concerns | n=20 smokers  n=20 never smokers  Gender: 9 male, 11 female  Age 28.7 (5.8)  Smoking history: 6.4 years (3.3)  Smoking status:11.1 (5.8) cig/day | Exposures: TC, ENDS.  9 puffs | 30 minutes  FMD | **Within subject: smokers** ENDS 5.88 (3.23) to 3.99 (2.02) *p*=0.017 TC 5.62 (2.96) to 2.82 (3.59) P=0.02  **ENDS vs. TC**: NS |
| Chaumont, 2019  Belgium  Non-profit medical organizations, pharmaceutical company, and private individuals funders | RCT – test 2  Randomized parallel arms  Hospital  High risk of bias | N=20  Gender: 17 male, 3 female  Age: 54 (2.0)  Smoking history**:** 34 (SD: 3) pack-years.  Smoking status: NR | 2 arms: n=10 non-nicotine ENDS  n=10 sham vaping  ENDS 17 ±1 puffs Sham vaping 15 puffs  non-nicotine, no flavor liquid prepared by hospital | 5, 20 minutes  HR | Non-nicotine ENDS vs Sham vaping  84(8) vs 77(2) *p*= 0.005 |
| Cobb, 2019  USA  Non-profit medical, government funders | Quasi-experimental  Cross-over  Location NR  High risk of bias | N=20  Gender: 10 male, 10 female  Age: 19.1 (1.1) | Exposures: TC, non-nicotine ENDS, ENDS  2 bouts of 10 puffs, start of test and at 60 minutes  36 mg/ml nicotine | Continuous 105 minutes  BP  HR | “No significant three-way interactions  for any measure.” |
| Frazen, 2018  Germany  University funder | RCT  Cross-over  Hospital  High risk of bias | N=15  Gender: 5 male, 10 female  Age: 22.9 (3.5)  Smoking history:  - smoking pack years:1-6 years; mean: 2.9 (SD: 1.5)  Smoking status: NR | Exposures: TC, non-nicotine ENDS, 24 mg/ml nicotine ENDS  10 puffs | 2 hours, testing every 5 minutes  peripheral SBP  peripheral DBP  central SBP  central DBP  HR | **ENDS vs. non-nicotine ENDS vs. TC: NS**  TC peripheral and central SBP higher than both ENDS at final test.  TC peripheral and central DBP higher than both ENDS at final test.  TC HR higher than both ENDS at final test. |
| Hiler, 2017  USA  Government funder | RCT  Clinical laboratory  High risk of bias | N= 64  Sub-group abstinence verified ENDS n=18 TC n=21  BL Gender: ENDS users: 27 male, 6 female TC users: 18 male, 13 female  BL N age: 30.8 (9.9)  Smoking history: NR  Smoking status: No. of cigarettes/d: 16.5 (9.4) | 2 arms: ENDS users, TC users. 4 nicotine strength conditions: 0 nicotine, 8 mg/ml, 18 mg/ml, 36 mg/ml nicotine  10 puffs, 2 sessions 60 minutes apart | HR monitored every 20 seconds, test results averaged for 5 minutes  HR | **Within subject** Collapsed groups mean  0 mg nicotine NS  8 mg/ml nicotine p<0.05 Session 1 66.4 (6.5) to 73.0 (7.4) S2 65.1 (6.5) to 69.9 (7.8)  18 mg/ml nicotine p<0.05 S1 66.4 (7.6) to 75.8 (7.9) S2 65.7 (7.7) to 71.6 (8.2)  36 mg/ml nicotine p<0.05 S1 66.6 (7.1) to 77.0 (8.6) S2 67.3 (7.8) to 72.7 (9.3)  **Between arms** ENDS vs. TC: NS |
| Kerr, 2019  UK  Medical non-profit funder | RCT  Cross-over  Location NR  High risk of bias | N=20  Gender: 20 male  Age: 31.6 (10.5)  Smoking history: NR  Smoking status: 7 (1-30) cig/day. | Exposures: ENDS, TC  15 puffs  18mg/ml nicotine | HR 1 minute SBP, DBP 10 minutes, 3 test mean | **Within-subject**  HR p<0.001  ENDS 65 (9) to 73 (8)  TC 64(8) to 86(13)  SBP, DBP: NS  **ENDS vs. TC**  HR: TC 23 (2.7) significant greater increase vs. ENDS 8 (1.2) p< 0.001.  SBP: TC significant greater increase 4 (9) vs. ENDS -1 (6) p=0.046 |
| Kuntic 2020  Germany  Non-profit organizations funder | Quasi-experimental  Location NR  High risk of bias | N = 20  Gender: 10 male, 10 female  Age 34.7 (10.2)  **Smoking history:** 11.6 ± 8.0 pack-years  **Smoking status:** 14 ± 5.03 cig/day. | Exposure: ENDS  40 puffs  18 mg/ml nicotine | 15 minutes FMD  10 minutes HR | **Within subject**  FMD decreased significantly p=0.017  HR increased p< 0.05 post-vaping (graph w/o exact measurement data) then decreased 10 minutes after vaping p<0.05 |
| Nides 2014  US  Industry funder | Quasi-experimental  Clinics  High risk of bias | N=25 at f/u  BL N Gender: 21 male, 12 female  BL N Age: 43 (18-63)  **Smoking history:** 21.1 years (2.46)  **Smoking status:** 20.1 (1.28) cig/day. | Exposure: ENDS  2 sessions of 10 puffs, 1 hour apart  Nicotine strength NR (26 mg in 0.5 ml cartridge) | 35 minutes  HR | **Within subject**  Test measurements NR (reported absolute change in beats per minute)  Session 1 NS  Session 2 significant increase 2.4 p<0.004 |
| Sumartiningsih, 2019  Indonesia  Commercial organization and university funders | RCT  Cross-over  High risk of bias | N=24  Gender: 24 male  Age: 23.2 (1.7)  **Smoking history**: 3.5±0.8 years.  **Smoking status**: average 9 cig/day | 3 exposures: non-nicotine ENDS, ENDS, TC  Puffs NR  3mg/ml nicotine | Immediate  SBP  DBP  HR | **Non-nicotine ENDS vs. ENDS vs. TC**  SBP NS  HR Non-nicotine ENDS 78 (12) vs. TC 85 (11) p=0.028  DBP Non-nicotine ENDS 76 (1) vs. 83 (8) p=0.017 |
| Szoltysek-Boldys, 2014  Poland  Government and university funders | Quasi-experimental  Cross-over  Medical university  High risk of bias | N=15  Gender: 15 female  Age: 23 (2)  **Smoking history:** 4±2 years.  **Smoking status:** 8±4 cigarettes per day. | 2 exposures: ENDS, TC  15 puffs  24 mg/ml nicotine | 10 minutes  DBP  SBP  HR | **Within-subject**  NS  **ENDS vs. TC**  Not analyzed |
| Vansickel, 2010  USA  Government funder | Quasi-experimental  Cross-over  Clinics  High risk of bias | N=32  Gender: 19 males, 13 females  Age: 33.6 (12)  **Smoking status:** mean 22 cigarettes per day (SD: 8.8) | Exposures: own TC, unlit TC, ENDS 18 mg nicotine cartridge, ENDS 16 mg cartridge  10 puffs 2 sessions 60 minutes apart | 45 minutes  HR | **Within-subject**  NS for ENDS  **TC vs. ENDS**  TC (over 65) higher than ENDS (under 65) p<0.05 |
| Walele 2016  UK  Industry funder | RCT  High risk of bias | N=12  Gender: 12 male  Age: 21-65  **Smoking history:** NR  **Smoking status:** 5–30 cig/day for at least one year | ENDS with flavored or unflavored liquid  10 puffs | 30 minutes after the 4^th^ test on day 4  SBP  DBP  HR  12-lead ECG | **All tests NS** |
| Yan, 2015  USA  Industry funder | RCT  Cross-over  High risk of bias | N=23  Gender: 11 male, 12 female  Age: 38.7 (10.77)  **Smoking status:** 10 or more manufactured cig/day | Exposures: TC and 5 ENDS products, 3 (A-C) at 24 mg/ml nicotine, 2 (D-E) at 16 mg/ml nicotine  2 exposures: 50 puffs; 1 hour ad lib use 49.5 – 60.3 puffs average, range 3-140 puffs | 20 minutes  SBP  DBP  HR | **Within subject**  SBP  Product D 118 (10.27) to 124 (12.46) p=0.02  Other ENDS NS  DSB  Product C 73 (8.61) to 76 (11.11) p=0.048  Product E 72 (7.21) to 76 (9.33) p=0.00017  Other ENDS NS  HR  Product B 71 (8.95) to 75 (8.63) p=0.008  Product C 70 (7.02) to 74 (7.14) p=0.002  Other ENDS NS  “considered to be not clinically significant”  **ENDS products vs. TC**  SBP NS  DBP Product C significantly less increase vs. TC p=0.048  DBP other ENDS vs. TC NS  HR NS |
| Follow-up |  |  |  |  |  |
| Cioe, 2020  USA  University and government funders | Quasi-experimental  High risk of bias | n=19 at f/u  N= 20 HIV-positive smokers  Gender: 14 male, 6 female  Age: 52.7 (9.3)  Smoking history: NR  Smoking status:TC/d: 15.1 (9.6) | Ad lib choice of flavors  Two ENDS models  18 mg/ml nicotine | 8 weeks  BP  HR | **Within-subject: NS**  Mean TC/day reduced 80% to 1.79 (2.2) n=7 TC abstinent |
| Cravo, 2016  UK  Industry funder | RCT  Parallel arms  Clinical testing centers  High risk of bias | N= 408 analyzed n=306 ENDS n=102 TC  n=287 ENDS completed study  n=40 subset 1 week confinement  Gender: ENDS: 168 male, 138 female TC: 58 male, 44 female  Age: 34.1-35.1 (10.1)  Smoking history: 5-30 cigarettes per day (CPD) for at least one year  Smoking status: Cigarette use ENDS arm 5-10/day 35.6% 11-20/day 56.2% 21-30/day 13.7% | 2 arms: ENDS supplied or TC own brand. TC abstinence requested, but no exclusion for non-compliance  2.0% nicotine | 1, 2, 4, 6, 8, 10 and 12-weeks follow up  SBP  DBP  HR  12-lead ECG | Test measurements NR “no clinically significant changes” |
| D’Ruiz, 2017  USA  Industry funder | RCT  Parallel arm  Confinement at research center  High risk of bias | N= 105  Gender: 68 male, 37 female  Age: 37.8 (11.1)  Smoking history: Mean years smoked: 18.8 (10.8)  Smoking status: Mean CPD: 18.8 (6.5) | 6 arms, n=15  3 arms exclusive ENDS, different models and flavors  2 arms dual use, different models. TC consumption reduced to 50% of BL use  1 arm abstinence  2.4%/24 mg/ml nicotine | 5 day study, measurements daily morning and evening  SBP  DBP  HR | **Between arms (ENDS, TC)** NS |
| Farsalinos, 2016  Italy  Health education non-profit funder.  12 week ENDS supplied gratis by manufacturer | RCT  Three-arms  Hospital  Some bias concerns | n=183 at f/u  n=145 “continuous smoking phenotype” sub-group at f/u  Gender sub group: 86 male, 59 female  Age: 41.6 (13.0) – 45.4 (14.4)  Smoking history:  -Median pack/yr: 24.9  Smoking status:Median cig/day:20.0 | 3 ENDS arms. 12 weeks ENDS supplied. Arm 1: 2.4% nicotine, Arm 2 :6 weeks 2.4% nicotine, 6 weeks 1.8% nicotine, arm 3 non-nicotine.  Post 12 weeks participants purchased and used ENDS as desired. | 2, 4, 6, 8, 12, 24, and 52 weeks  SBP  DBP  HR | **Within subject**  SBP 128.0 (15.3) to 123.1 (13.8) p=0.004 “slight decrease”  Other tests NS  **Sub-group**  SBP 126.0 (15.6) to 122.6 (13.3) p=0.001 “small decrease” participants with normal SBP at BL NS  participants (n=66) with elevated SBP at BL 141.2 (10.5) to 132.4 (12.0) p<0.001  DBP 76.7 (9.9) to 75.2 (9.4) p=0.02 “small reduction”  HR NS  TC abstinent no ENDS use vs TC abstinent ENDS users  BP NS |
| George, 2019  UK  Medical non-profit, university, and government funders. | RCT  High risk of bias | n=114 at f/u  Recruitment N = 145  End of study TC n=40, 13 male ENDS n=37, 14 male non-nicotine ENDS n=37, 12 male  Age: TC 44.2 (40.4 – 47.9) ENDS 48.0 (44.7 – 51.3) non-nicotine ENDS 48.4 (43.5-53.3)  Smoking history: Years smoked: TC 29.0 (19.5-36.5); EC-Nicotine 36.0 (25.0-41.0); EC- Nicotine free 32.0 (22.0-40.0).  Pack-year history: TC 25.4 (15.5-36.5); EC-Nicotine 33.3 (21.8-44.0); EC- Nicotine free 27 (19.9-36.8)  Smoking status: Cig/day: TC 20 (15-20); EC-Nicotine 18 (15-20); EC- Nicotine free 18 (15-20). | 3 arms: ENDS, non-nicotine ENDS. TC arm: participants preferring not to stop TC use.  Nicotine16 mg Non-nicotine with nicotine flavoring  Noncompliant dual use n=9 ENDS 0.52 TC/day n=10 ENDS 2.34 TC/day n=13 non-nicotine ENDS 3.22 TC/day n=6 non-nicotine ENDS 10.53 TC/day | 4 week follow up  HR  SBP  DBP  FMD | **Between arms** HR Participants < 20 pack-years ENDS vs. TC increased 2.6 (0.3-5.0) non-nicotine ENDS vs. TC increased 5.2 (0.6-10.0) p=0.03  Participants >20 pack years ENDS vs. TC decreased 2.8 (-5.2-0.4) non-nicotine ENDS decreased 5.6 (-10.4-0.8) p=0.02  SBP, DBP: NS adjusted for BL variables  FMD Females ENDS vs. TC change 1.824 (0.942-2.706) p<0.0001 Males NS Females non-nicotine ENDS vs. TC change 2.183 (1.33-3.030) p<0.0001 Males NS  ENDS vs. non-nicotine ENDS: NS |
| Hickling, 2018  UK  Non-profit charity, non-profit medical, university | Quasi-experimental  Within-subject  Location NR  High risk of bias | n=40 at f/u  Gender: 38 male, 12 female recruited  Age: 38.96 (10.73)  Serious mental illness | TC reduction, 6 weeks ENDS supplied  4.5% nicotine | BL, weekly 1-10 weeks, and 24 weeks  HR  BP | HR test measurements NR  **Within subject**  BP BL to 6 weeks test measurements similar |
| Ikonomidis, 2020  Greece  No funder | RCT  Clinic  Some concerns | N=40  Gender: 8 male, 32 female  Age: 46.8 (10.9)  Smoking history: NR  Smoking status: 25.8 ± 9.2 conventional cigarettes per day | 2 arms: n=20 ENDS, n=20 continued TC use  12 mg/ml nicotine | 4 months  SBP  DBP | **Within subject** NS  **Between arms** analysis not performed. |
| Polosa 2016  Italy  University funder | Cohort  Outpatient clinics  Some concerns | N = 89, hypertension  ENDS = 43  Gender: 26 male, 17 female  Age: 53.5 (6.3)  Sub-group Exclusive ENDS = 21, 11 male, 10 female  Sub-group Dual users = 15 male, 7 female  Control = 46  Gender: 24 male, 22 female  Age: 54.2 (7.5)  **Smoking status:** 21.5 (6.9) | Patients reporting regular daily use of ENDS on at least two consecutive follow up visits after smoking at BL  Matched controls on 8 factors at BL | 6, 12 months  SBP  DBP  HR | **Within subject ENDS**  SBP  140 (134.5,144) to 130 (123.5, 138.5) p<0.001  DBP  86 (78, 90) to 80 (74.5, 90) p=0.006  HR: NS  **ENDS vs. Control**  SBP 130 (123.5, 138.5) vs. 145 (136.3, 150) p<0.001  DBP 80 (74.5, 90) vs. 85 (85, 90) p<0.001  HR NS  **Exclusive ENDS users vs. Dual Users** (Dual users averaged 80% reduction in TC/day)  SBP and DSB significant reduction vs. SBP significant reduction, DBP NS |
| Van Staden, 2013  South Africa  Industry funder | Quasi-experimental  High risk of bias | n=13 at f/u  N = 15  Gender: 8 males, 5 females  Age: 38 (23 – 46) years  **Smoking history:** median 17 years (range 5 - 27)  **Smoking status:** median: 20 cigarettes (range 10 - 30) | ENDS: Twisp e-cigarette  0.8 ml nicotine | 2 weeks  HR  BP | Test data NR  Reported results as NS |
| Veldheer, 2019  USA  Government funder | RCT  4 arms  2 university sites  Some bias concerns | n=263 analyzed  n=191 ENDS  Gender: 56.5% female  Age 46.5 (11.4)  **Smoking status:** mean baseline cig/day: 19.1 (7.2) for cig-sub group/18.1 (6.5) for e-cig group | 4 arms: cigarette substitute, non-nicotine ENDS, 8 mg/ml nicotine ENDS, 36 mg/ml nicotine ENDS  “Encouraged” to reduce smoking by 50% for 2 weeks, then 75% for two weeks, and maintain reduction. | BL, 1, 3 months  SBP  DBP  HR | All tests NS |
| Walele 2018  UK  Industry funder | Quasi-experimental  2 clinical research centers  High risk of bias | n= 206 analyzed  n=102 completed study  Gender: 115 male, 94 female  Age: 36.6 (10.2)  **Smoking history:** NR  **Smoking status:** 5–30 cig/day for at least one year | 16 mg/ml nicotine  Participants “reminded” to use only study ENDS; not terminated for TC use | BL, 1, 2, 3, 6, 9, 12, 15, 18, 21, 24 months  SBP  DBP  HR  ECG | **Within-subject**  “No clinically relevant, product-related findings were observed for vital signs, ECG.” |

BL: Baseline; DBP: Diastolic blood pressure; ECG: Electrocardiogram; ENDS: Electronic nicotine delivery systems; FMD: Flow-mediated dilation; NR: Not reported; NS: Not significant;; RCT: Randomised Controlled Trial; S#: session number; SBP: Systolic blood pressure; TC: Tobacco cigarette.

**Online Resource 11 Study biases**

| Study  Bias Rating | Design | JBI | JBI blinding items | Other JBI Items | Potential Sources of Bias  *Reporting biases in italics* |
| --- | --- | --- | --- | --- | --- |
| Biondi-Zoccai  High Risk of Bias | RCT  Acute  Cross-over | 7/10 | Allocation unclear.  No participant blinding.  No treaters blinding. | None | Substantial deviations from protocol.  Small sample size; no power calculation.  Wash-out period compliance questionable.  *Reporting: Data discrepancies in text.*  *Spin: NS findings discussed as ENDS less detrimental.* |
| Carnevale  Mastrangeli  Some Concerns | Quasi-experimental  Acute | 8/8 | NA | None | No protocol.  No concealment.  Small sample size without power calculation. |
| Chaumont  High Risk of Bias | RCT  Acute effects | 10/13 | Allocation not concealed.  No participant blinding.  No treaters blinding. | None | Protocol deviations.  Excessive ENDS exposure.  Small sample size, power calculation inferred.  Ascertainment bias: heavy smokers.  *Reporting: Substantial and multiple discrepancies between data and discussion.*  *One-sided reference: anti-ENDS, animal studies.*  *Spin: over-generalization.* |
| Cioe  High Risk of Bias | Quasi-experimental | 7/9 | NA | No control group.  Drop-outs not analyzed. | No protocol.  High drop-out rate not analyzed.  Small sample size, no power calculation.  *Vital signs not emphasized in conclusion. No discussion of other cv studies.* |
| Cobb  High Risk of Bias | Quasi Experimental  Acute  Cross-over | 5/6 | NA | Statistical analysis for cardiovascular unclear. | No published protocol.  Test measurements NR.  Very high nicotine content ENDS.  Small sample size, no power calculation.  Potential volunteer bias: high compensation.  Ascertainment bias: very young adults.  *Reporting: no discussion of cardiovascular data.* |
| Cravo  High Risk of Bias | RCT | 9/13 | Allocation not concealed.  No participant blinding.  No treaters blinding.  Assessors blinding unclear. | No additional items. | Only narrative reporting of cardiovascular tests.  Multiple protocol discrepancies, potential data dredging.  High rate of treatment non-compliance. *Data discrepancy between LDL results and discussion.*  *One-sided references. Spin: emphasis on NS results.* |
| D'Ruiz  High Risk of Bias | RCT | 6/13 | Allocation unclear.  No participant blinding.  No treaters blinding.  Assessors blinding unclear. | Randomization unclear.  Treatment groups dissimilar. | Substantial differences between protocol and study outcome measurements, possible data dredging.  Potential volunteer bias: compensation NR.  *Reporting: Tables 6 and 8 incorrectly formatted.* |
| Farsalinos 2016  Some Concerns | RCT | 12/13 | Assessors blinding unclear. | None | Deviations from protocol, post-hoc analysis acknowledged by authors. |
| Franzen 2018  High Risk of Bias | RCT  Acute  Cross-over | 9/10 | None | Analysis issues: inconsistent attribution of significance. | Deviations from protocol.  Specific tests measurements NR.  Small sample size.  Ascertainment bias: exclusively young participants.  *Reporting: multiple data discrepancies.*  *Spin: mid-trial significant measurements represented as final outcomes which were NS. Significant reduction in negative effects of ENDS vs. TC not discussed.* |
| George 2019  High Risk of Bias | RCT | 10/13 | Participant blinding unclear (nicotine/non-nicotine).  Treater blinding unclear. | Drop-outs not compared. | Substantial non-compliance, not all statistical analyses accounted for non-compliance.  High drop-out.  Surrogate marker for CV outcomes.  *Spin bias: NS results reported as improvement.* |
| Hickling  High Risk of Bias | Quasi-experimental | 7/9 | NA | No control group. Follow-up incomplete. | Deviations from protocol for primary and secondary outcomes.  Substantial reduction in tests from protocol.  Test data NR.  Small sample size.  *Reporting: no discussion of cardiovascular data. No details on recruitment.* |
| Hiler  High Risk of Bias | RCT  Acute | 8/12 | Allocation unclear.  Assessor blinding unclear. | Randomization unclear.  Treatment groups similarity unclear. | Protocol not published.  No power calculation.  *Reporting: No discussion of HR results. HR not included in the conclusion.* |
| Ikonomidis  Some Concerns | RCT | 10/13 | No participant blinding.  Treater blinding unclear.  Assessors blinding unclear. | None | Protocol not published.  No power calculation.  Ascertainment bias: 80% female participants. Heavy smokers. |
| Kerr  High Risk of Bias | RCT  Acute  Cross-over | 6/10 | No allocation concealment.  No participant blinding.  No treater blinding.  Assessors blinding unclear. | None | No protocol.  Small sample size.  Ascertainment bias: all male.  *Reporting: discrepancy between text and figure 3a.* |
| Kuntic  High risk of bias | Quasi-experimental  Acute | 7/8 | NA | No control group | No protocol.  Excessive ENDS exposure.  No TC abstinence before testing.  Small sample size.  *HR findings not discussed. No discussion of other human studies. Study focused on mouse testing.* |
| Nides  High Risk of Bias | Quasi-experimental  Acute effects | 5/8 | NA | No control group.  Drop-outs not analyzed.  Statistical analysis issues: group means but high variation in participant results; beats per minute changes for data analysis. | Test measurements NR.  No discussion of cardiovascular results.  Power calculation issues.  Potential volunteer bias: compensation.  *Reporting: HR figure does not match narrative description.* |
| Polosa  Some Concerns | Cohort  Hypertension | 9/10 | NA | Incomplete strategy for one of 5 confounders. | No published protocol.  Abstinence self-report.  *Reporting: 1 p value incorrect (Control DBP),* |
| Sumartinigsih  High Risk of Bias | RCT  Acute effects | 5/11 | Allocation unclear.  Treater blinding unclear.  Assessor blinding unclear. | Drop-outs not analyzed.  Analysis missing participants.  Outcome measurements not reliable (wide range). | No published protocol.  No verification of abstinence.  ENDS exposure not reported.  No power calculation.  Ascertainment bias: all young male athletes.  *Reporting: major difference in p values significance reported between table 4 and text.*  *Spin: emphasis on NS results.* |
| Szoltysek-Boldys  High Risk of Bias | Quasi-experimental  Acute effects. | 6/7 | NA | Dissimilar participants: wide variation in smoking history. | No protocol.  No verification of pre-testing abstinence.  Small sample size.  High nicotine content.  Ascertainment bias: all female, young adults, light smoking. |
| Van Staden 2013  High Risk of Bias | Quasi-experimental | 7/9 | NA | No control group.  Participants lost to follow-up not sufficiently analyzed. | No protocol identified.  Only narrative reporting for cardiovascular tests.  Small sample size; power calculation issues.  High drop-out.  No details on exclusion criteria. |
| Vansickel  High Risk of Bias | Quasi-experimental  Acute | 7/8 | NA | Statistical analysis issues. | No published protocol.  Substantial indirectness of ENDS: 1^st^ generation models did not produce measurable nicotine exposure.  Potential data dredging: “all data combined.” |
| Veldheer 2019  Some Concerns | RCT | 12/13 | No issues. | Not all participants completed all testing. | Deviations from protocol.  One arm high nicotine ENDS. |
| Walele 2016  High Risk of Bias | RCT | 8/13 | No allocation concealment.  No participant blinding.  No treater blinding.  Assessors blinding unclear. | Participants lost to follow-up not analyzed. | Protocol discrepancies.  Only narrative reporting for cardiovascular tests.  Small sample size. |
| Walele 2018  High Risk of Bias | Quasi-experimental | 6/9 | NA | No control group.  Recruitment from prior RCT included participants with and without ENDS exposure.  Mixed participants in group analyses. | Protocol discrepancies: change in designation of primary outcomes.  Potential spill-over effect from prior RCT.  Low compliance on TC abstinence (53.4% with 80% compliance).  Single investigator rating of adverse events.  *Spin: emphasis on secondary outcome.* |
| Yan 2015  High Risk of Bias | RCT  Acute | 6/10 | Allocation unclear.  Participant blinding unclear.  Treater blinding unclear.  Assessor blinding unclear. | None | No published protocol.  No verification of wash-out abstinence.  Excessive measured ENDS exposure.  Highly variable ad lib ENDS exposure  *Reporting: multiple discrepancies within text and with tables and figures on significance of test results.*  *Spin: over-emphasis on selected results.* |

Online Document 12 GRADE calculation

| Study | Study design | Lowered for bias | Evidence issues |
| --- | --- | --- | --- |
| Biondi-Zoccai [27] | Randomized trial | High -2 |  |
| Carnevale [28]  Mastrangeli [29] | Randomized trial | Some concerns - 1 | ENDS vs TC not significant |
| Chaumont [30] | Randomized trial | High - 2 | Imprecision -1 |
| Cioe [31] | Randomized trial | High -2 |  |
| Cobb [32] | Randomized trial | High -2 |  |
| Cravo [33]* | Randomized trial | High - 2 | Indirectness - 1 |
| D’Ruiz [34]* | Randomized trial | High -2 |  |
| Farsalinos [35] | Randomized trial | Some concerns - 1 | Only hypertension subgroup significant |
| Frazen [36] | Randomized trial | High - 2 |  |
| George [37] | Randomized trial | High -2 |  |
| Hickling [38] | Randomized trial | High -2 |  |
| Hiler [39] | Randomized trial | High -2 |  |
| Ikonomidis [40] | Randomized trial | Some concerns - 1 | No significant effects |
| Kerr [41] | Randomized trial | High -2 |  |
| Kuntic [42] | Randomized trial | High -2 | Imprecision - 1 |
| Nides [43]* | Randomized trial | High - 2 | Indirectness - 1 |
| Polosa [44] | Observational | Some concerns - 1 |  |
| Sumartiningsih [45] | Randomized trial | High - 2 |  |
| Szoltysek-Boldys [46] | Randomized trial | High - 2 | Indirectness - 1 |
| Van Staden [47]* | Randomized trial | High - 2 | Indirectness - 1 |
| Vansickel [48] | Randomized trial | High - 2 | Indirectness - 1 |
| Veldheer [49] | Randomized trial | Some concerns - 1 | No significant effects |
| Walele 2016 [50]* | Randomized trial | High -2 |  |
| Walele 2018 [51]* | Randomized trial | High - 2 | Imprecision - 1 |
| Yan [52]* | Randomized trial | High - 2 |  |
